# Diabetic foot disease management in the Gulf Cooperation Council countries: a scoping review protocol

**DOI:** 10.1093/epirev/mxaf012

**Published:** 2025-07-18

**Authors:** Mariam Alessa, Rhonda Clifford, Kevin Murray, Barbara Nattabi, Houssam K Younes, Deborah Schoen

**Affiliations:** The University of Western Australia, Crawley WA, Australia; Ministry of Health Kuwait, Kuwait City, Kuwait; The University of Western Australia, Crawley WA, Australia; The University of Western Australia, Crawley WA, Australia; The University of Western Australia, Crawley WA, Australia; Cleveland Clinic Abu Dhabi, Abu Dhabi, United Arab Emirates; The University of Western Australia, Crawley WA, Australia

**Keywords:** Arab Gulf states, diabetes, diabetic foot complications, lower extremity amputations, peripheral artery disease

## Abstract

The Gulf Cooperation Council (GCC) countries face a substantial impact from the increasing prevalence of diabetes mellitus, which experts identify as a major public health challenge in the region. Despite the escalating burden of diabetes mellitus and its related complications, including diabetic foot disease (DFD), there are noteworthy knowledge gaps concerning the prevalence and trends of DFD in the GCC countries. Furthermore, there is insufficient understanding of the management of DFD within health care settings in this region. The objective of this scoping review is to comprehensively assess the extent and nature of DFD management across different health care settings in GCC countries. The study will use the population, concept, and context framework: the population of interest is individuals with DFD, the concept is the management or treatment of DFD or its complications, and the context includes the GCC countries. The review will include published articles and unpublished quantitative and qualitative research papers, from 1981 onward, aligning with the establishment of the first multidisciplinary team diabetic foot clinic at King’s College Hospital, London. The scoping review will follow guidelines from Joanna Briggs Institute (JBI) and be reported following the Preferred Reporting Items for Systematic Review and Meta-Analyses extension for Scoping Reviews Checklist. A comprehensive search will be conducted, across various databases including CINAHL, MEDLINE (Ovid), Embase, Scopus, Cochrane CENTRAL, PsycINFO, Global Health, and the Arabic database Al Manhal, and gray literature sources. Studies in Arabic and English language will be included. A data extraction tool will be used to extract the data and will enable a chronological narrative synthesis of results.

## Introduction

Diabetes mellitus (DM) is a global public health concern, and diabetic foot disease (DFD) is 1 of the 4 major complications affecting individuals and healthcare systems.^1^ More than 500 million people worldwide have DM, making up over 10% of the adult population.^2^ Every 7 seconds, someone dies of a DM-related complication.^3^ Diabetes mellitus is now the leading cause of kidney failure and lower extremity amputations (LEA) globally.^4^ An amputation is performed somewhere in the world on a person with DM every 20 seconds.^5^ The risk of a person with DM having a LEA is estimated to be 23 times that of a person without DM.^6^ This occurs because individuals with DM face a markedly increased risk of disabling and limb-threatening foot disease, with the likelihood of this condition arising during their lifetime ranging from 19% to 34%.^7^ Several risk factors, including loss of protective sensation, peripheral arterial disease, foot deformity, history of foot ulceration, and previous LEA,^8^ increase the likelihood of DFD,^9^ with adverse effects on morbidity, death, and quality of life.^5^^,^^10–13^

According to the International Diabetes Federation (IDF), approximately 73 million individuals, accounting for 1 in 6 adults, are affected by DM in the Middle East and North Africa region. This prevalence marks the highest proportion among all the IDF Regions.^2^ The Gulf Cooperation Council (GCC) within the Middle East and North Africa region comprises Bahrain, Kuwait, Oman, Qatar, Saudi Arabia, and the United Arab Emirates (UAE), totaling an estimated population of 53 million.^14–16^ A retrospective study^17^ revealed that the GCC countries exhibit a notably higher prevalence of 24%, compared with 16% in non-GCC nations such as Lebanon, Cyprus, Iraq, Jordan, Palestine, Turkey, Syria, Egypt, Iran, and Yemen.

**Figure 1:**
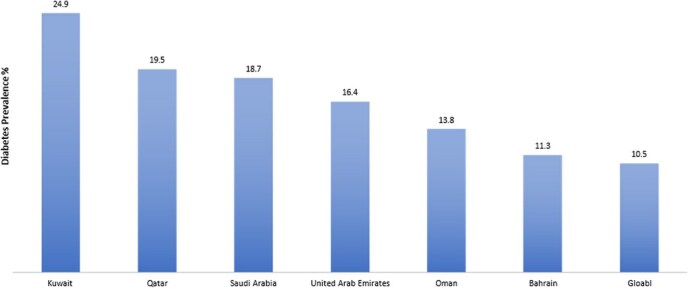
Summary of global and country-level diabetes prevalence estimates for 2021 (adapted from the International Diabetes Federation^2^).

The GCC countries, characterized by similar sociodemographic features and health care systems, have witnessed rapid economic growth and urbanization. These changes have led to more sedentary lifestyles, contributing to an increased prevalence of obesity and noncommunicable diseases, including DM.^15–18^ Despite stable obesity rates, the prevalence of DM is projected to increase by 96% in the GCC countries by 2035.^8^^,^^19^ Each GCC country has a higher prevalence of DM in adults compared with global rates (Figure 1)^2^; according to the IDF, DM affects 1 in 5 residents in GCC countries, underscoring its significance as a major health issue.^14^^,^^15^^,^^20^^,^^21^

Citizens of the GCC and Arab residents with DM, regardless of their religion, face unique cultural and traditional barriers to foot care that put them at greater risk for DFD. The use of complementary or alternative medicine, seeking the advice and treatment from herbalists to heal gangrene, and attending traditional healers for cautery and bloodletting are still common practices.^20^ Misconceptions about DM and its complications are also prevalent.^22^ Moreover, socioeconomic factors like poverty, walking barefoot, unsanitary conditions, and specific cultural or religious customs can exacerbate the vulnerability to foot injuries.^5^^,^^23–25^

Despite the escalating burden of DM and its associated complications in GCC countries, there are notable knowledge gaps regarding the prevalence and trends of DFD. Although the prevalence of diabetic foot problems is expected to be high in the GCC countries,^26^ only Saudi Arabia has published incidence studies,^22^ and 17 of the 22 Arab countries have not reported prevalence rates of diabetic foot ulcers. Additionally, there are deficiencies in the available care and management options across various health care settings, exacerbating the challenges associated with this condition.^14^ Health care systems in these regions have yet to adapt to the increasing DM burden,^14^^,^^18^^,^^27^ resulting in suboptimal diabetes care and related complications management.^15^^,^^18^ One study conducted in the UAE found that 72% of patients with DM lack access to crucial foot care, which is a cause for concern given that the UAE has 1 of the highest DM burdens in the Middle East (16.3%).^22^^,^^24^ This underscores the pressing need for further research and health care system enhancements to address this public health issue. Diabetic foot disease places a significant financial burden on health care systems,^10^^,^^28^ with a substantial portion of expenditures allocated to its treatment.^12^ A study conducted in Australia highlighted DFD as a prominent complication of DM, ranking second in its impact on health services but receiving comparatively minimal funding.^1^ Providing optimal care necessitates evidence-based guidance for health care professionals.^7^^,^^8^^,^^29^ However, there remains a dearth of evidence regarding the implementation of these guidelines for managing DFD within GCC countries. This lack of knowledge extends to the status of diabetic foot health services across the GCC region. For example, podiatry care services remain scarce, with just over a dozen certified podiatrists employed in the Middle East in 2009 based on the records of the International Working Group on the Diabetic Foot (IWGDF).^30^ No recent data or updates are available on the number of current practicing podiatrists in the Middle East.

Enhancing the management of DFD conditions across diverse health care settings holds the key to significantly improving the well-being of affected individuals^14^^,^^31^ and is an important aspect of health system performance.^32^ Given the escalating burden of diabetes in GCC countries and the lack of substantial knowledge on DFD and its management in this specific context, an urgent need arises for a literature review on this subject. The aim for this review is to map existing knowledge and illuminate gaps. Optimal for this purpose, a scoping review provides a robust foundation for exploring a broad spectrum of diverse data.^2^^,^^16^^,^^17^ Notably, a recent search across the MEDLINE, the Cochrane Database of Systematic Reviews, and JBI Evidence Synthesis databases revealed no evidence of any ongoing or completed relevant systematic or scoping reviews on this topic.

### Research question

The objective of this scoping review is to assess the nature and extent of DFD management across services at primary, secondary, and tertiary health care services in GCC countries. Our primary research question asked: what is the epidemiologic landscape of DFD and how is it managed in the GCC countries? It was based on the population, concept, and context elements of inclusion criteria.^32^ Within that overall question, we had the following secondary questions: What are the sociodemographic characteristics and clinical outcomes for patients with DFD in GCC countries? What are the risk factors influencing DFD in GCC countries? What are the reported characteristics of foot ulcers in individuals treated for DFD? What are the different clinical settings (eg, primary, secondary, and tertiary care; community vs hospital-based care) providing DFD care in GCC countries? And, what are the barriers to and facilitators of quality diabetic foot care in GCC countries?

## Methods

### Eligibility criteria

#### Participants

The eligibility criteria for this study were guided by the population, concept, and context framework.^33^ The concept is management (including education and prevention) or treatment of DFD. The context is within GCC countries. Participants in the studies are adults from any racial and ethnic background. In this context, the population under consideration is specifically defined as individuals with DFD. We adhered to the IWGDF’s definition of DFD: “Disease of the foot of a person with current or previously diagnosed diabetes mellitus that includes one or more of the following: peripheral neuropathy, peripheral artery disease, infection, ulcer(s), neuro-osteoarthropathy, gangrene, or amputation.”^34^^,^^35^

#### Types of sources

In this scoping review, we consider both experimental and quasi-experimental study designs meeting the inclusion criteria and aligning with the research question, including randomized controlled trials, nonrandomized controlled trials, before-and-after studies, and interrupted time-series studies. Additionally, analytical observational studies, including prospective and retrospective cohort studies, case-control studies, and analytical cross-sectional studies, will be considered for inclusion. Descriptive observational study designs, such as case series, individual case reports, and descriptive cross-sectional studies, will also be considered. Qualitative studies focusing on qualitative data, using designs like phenomenology, grounded theory, ethnography, qualitative description, and action research, will be part of the review.

## Methods

This scoping review will be conducted in accordance with the guidelines from the JBI^32^ and reported according to the Preferred Reporting Items for Systematic Review and Meta-Analyses extension for Scoping Reviews (PRISMA-ScR) Checklist.^36^

### Search strategy

The search strategy aims to encompass both published and unpublished studies. Initially, a limited search of MEDLINE, CINAHL, and topic-based search filters developed by Flinders University was conducted to identify relevant articles and keywords on the topic. The text words present in the titles and abstracts of pertinent articles, along with the index terms used to describe the articles, were used to formulate a comprehensive search strategy for MEDLINE (Ovid) (Table 1). This search strategy, inclusive of all identified subject headings, key words, and index terms, will be adjusted for other databases and/or information sources using the Systematic Review Accelerator (SR-Accelerator) Polyglot search. This free software, developed at Bond University, facilitates the translation of a MEDLINE/PubMed search strategy to other major databases.^37^ Gray literature will be searched within the references of identified articles and in gray literature databases using a simplified search strategy of combining key words using Boolean operators where possible. Studies published in Arabic and English will be included. Studies published since 1981 will be included as this decision aligns with the establishment of the first multidisciplinary team diabetic foot clinic and team at King’s College Hospital in May 1981 by Mike Edmonds.^38^

**Table 1:** Search strategy for Ovid MEDLINE: ALL <1981 to May 1, 2024>.

**Search no.**	**Syntax**	**Results from May 23, 2024**	**Domain**
1	exp Diabetes Mellitus/	526 217	Population^a^: people with diabetic foot disease
2	diabet^*^.mp. ^*^	882 268	
3	1 or 2	884 934	
4	exp Foot Diseases/or exp Foot/or exp Foot Deformities/or exp Diabetic Foot/or exp Foot Orthoses/ or exp Foot Ulcer/	91 112	
5	(Foot or feet or limb^*^ or Extremit^*^ or LEA^*^ or LLA^*^ or leg^*^ or peripheral).mp.	5 932 706	
6	complications.mp. or exp Diabetes Complications/	3 533 925	
7	4 or 5 or 6	8 839 189	
8	exp Pain Management/ or exp Change Management/or exp Self-Management/or exp Medication Therapy Management/or exp Disease Management/or exp Case Management/or exp Risk Management/or exp Financial Management/	5 241 822	Concept: management of diabetic foot disease or its complications
9	(Management or treat^*^).mp.	8 542 458	
10	8 or 9	8 765 343	
11	exp Middle East/	167 604	
12	(“Middle east” or Bedouin^*^ or Arab^*^ or GCC or gulf or Persian or kuwait or qatar or saudi arabia or KSA or oman or “united arab emirates” or UAE or “abu dhabi” or dubai).mp.	254 154	Context: GCC countries
13	11 or 12	376 465	
14	3 and 7 and 10 and 13	2749	Result

Abbreviation: GCC, Gulf Cooperation Council.

a [mp = title, book title, abstract, original title, name of substance word, subject heading word, floating sub-heading word, keyword heading word, organism supplementary concept word, protocol supplementary concept word, rare disease supplementary concept word, unique identifier, synonyms, population supplementary concept word, anatomy supplementary concept word].

The databases to be searched are CINAHL complete, MEDLINE (Ovid), Embase, Scopus, PsycINFO, Global Health, and the Arabic database Al Manhal. Sources of unpublished studies or gray literature will include Google Advanced, E-marefa, and GCC government websites including those of health departments (Table 2).

**Table 2:** List of databases to be searched.

**For published articles**	**For gray literature**
MEDLINE (Ovid)	Google advanced
Scopus	GCC governmental websites: Secretariat General of GCC World Bank, GCC Cooperation Council for the GCC Gulf Cooperation Council Global Edge: GCC Recourses GCC-STAT (https://gccstat.org/en/) GobalEdge: GCC (https://globaledge.msu.edu/trade-blocs/gcc) Middle East Policy Council (https://mepc.org/) Ministry of Health & Prevention, United Arab Emirates (https://mohap.gov.ae/en/home) Ministry of Health, Kuwait (https://www.moh.gov.kw/en/Pages/default.aspx) Ministry of Public Health, Qatar (https://www.moph.gov.qa/english/Pages/default.aspx) Ministry of Health, Sultanate Oman (https://www.moh.gov.om/en/) Ministry of Health, Kingdom of Saudi Arabia (https://www.moh.gov.sa/en/Pages/default.aspx) Ministry of Health, Kingdom of Bahrain (https://www.moh.gov.bh/?lang=en) Department of Health, Abu Dhabi (https://www.doh.gov.ae/en/) Abu Dhabi Health Services Company PJSC (https://www.seha.ae/) Statistics Center Abu Dhabi (https://scad.gov.ae/) General Authority for Statistics, Kingdom of Saudi Arabia (https://www.stats.gov.sa/en) Dubai Statistics Centre (https://www.dsc.gov.ae/en-us/Pages/default.aspx) Kuwait General Statistics Bureau (https://www.csb.gov.kw/Pages/Statistics_en?ID=67–ParentCatID=1) Bahrain Open Data Portal (https://www.data.gov.bh/pages/homepage/) Oman National Centre for Statistics and Information (https://omanportal.gov.om/wps/wcm/connect/en/site/home/gov/gov1/gov5governmentorganizations/ncfsi/ncfsi) Qatar Planning and Statistics Authority (https://www.psa.gov.qa/en/Pages/default.aspx)
CINAHL complete	E-Marefa: https://emarefa.net/
PsycINFO	
Embase	
Al Manhal	
Global Health	

Abbreviation: CINAHL, GCC, Gulf Cooperation Council.

Searches will be conducted using English search terms only. However, Arabic-language studies will still be included in the review if they appear in the search results, through reference list screening, or from gray literature sources. This approach balances comprehensive coverage with methodological feasibility. The potential limitation of missing Arabic-only indexed studies is acknowledged and will be addressed in the review’s discussion.

### Study or source of evidence selection

After the initial search, all identified citations will be exported into EndNote.^39^ In the subsequent duplicate removal procedure, any identical references will be systematically eliminated. The title and abstract screening stage will be undertaken in Covidence.^40^ Titles and abstracts will be reviewed for alignment with the predefined inclusion criteria by a primary reviewer proficient in both Arabic and English. A second reviewer will independently screen titles and abstracts. Discrepancies will be resolved by discussion or a third reviewer of the titles and abstracts. Full-text articles will then be assessed using the same process.

Transitioning to the full-text stage, articles will be retrieved in full and their citation details will be imported into Covidence.^40^ Sources of evidence that do not meet the inclusion criteria at the full-text stage will be comprehensively documented, ensuring transparency in the scoping review process.

This comprehensive approach, encompassing duplicate removal, meticulous title and abstract screening in Covidence,^40^ and rigorous full-text assessment, ensures an objective and thorough evaluation of the included sources in the scoping review.

### Data extraction

Data extraction will be performed by 1 reviewer using a data extraction tool developed by the reviewers and verified by a second reviewer to ensure accuracy and consistency. This tool, adapted from a report by Jeffcoate et al.,^41^ also integrates insights from Campbell et al.,^42^ in turn expanding upon Donabedian’s model^43^ for a comprehensive understanding and assessment of health care quality; however, it also includes the concept of patient-centered access to health care proposed by Levesque et al.^44^ This combination broadens the tool’s scope, enabling a holistic evaluation that considers not only clinical markers of good quality but also aspects of accessibility and patient-centricity within diabetic foot care delivery. The extracted data will encompass specific details about participants, the concept under study, the context, study methods, and key findings relevant to the review questions.

A draft of the extraction form is provided (Table 3). Any discrepancies among reviewers will be resolved with the intervention of a third reviewer. If deemed appropriate, authors of the articles and papers may be contacted to request missing or additional data, as required. All modifications and any communications with authors will be thoroughly documented in the scoping review.

**Table 3:** Data extraction instrument.

**Main category**	**Description**
Authors	
Title	
Journal	
Year of publication	
Type of study	Survey/questionnaire/cross-sectional study/medical records/physical examination
Study population	Hospital-based/area-based/clinic-based/community-based/hospital discharge data/annual estimates of civilians, noninstitutionalized, hospitalized individuals
Dropout rate	
Geographic location	
Prevalence of diabetes in geographic location	
Structure	
Health care setting of study	What is the setting of the study? What is the rationale for choosing this setting? Is sufficient detail given about the setting? Over what period is the study conducted?
Material resources	Place of delivery The adequacy of the physical environment Availability of necessary equipment
Human resources	Staffing levels Qualifications of health care professionals
Phenomenon under study (if qualitative)	What is being studied? Is sufficient detail given of the nature of the phenomenon under study?
Outcomes of qualitative studies	What outcome criteria are used in the study? Whose perspectives are addressed (professional, service, user, carer)?
Population characteristics	
Individuals	Age, sex, and ethnicity Diabetes type, duration, and adequacy of glycemic control Relevant cardiovascular drugs Immunosuppression Comorbid conditions (eg, established renal failure, heart failure, immobility, impaired vision, coronary artery disease, heart failure, cerebrovascular disease, renal disease, depression) Ulcer risk classification IWGDF: very low, low, medium, or high Ambulatory status Educational status, socioeconomic status, and capacity for self-care (for studies on education) Smoking status Previous interventions for peripheral artery disease
Limb	Peripheral artery disease: minimal assessment by palpation of pulses and ankle-brachial pressure index, toe blood pressure, or both Neuropathy: minimal assessment by determining loss of protective sensation (eg, with a 10 g monofilament or vibration perception or Ipswich Touch test) Vascular imaging: if any further vascular imaging done Limb symptoms: none, atypical (weakness or limping), intermittent claudication, and rest pain Toe systolic pressure, toe-brachial pressure index, or TcPo_2_ Arterial pulse waveform Anatomic distribution of the vascular disease in the leg Foot deformity (type or severity, or both) History of previous foot ulceration History of amputation Indication Amputation level The rationale for amputation level Amputation definition No. of patients/extremities/procedures (re-amputation)
Ulcer	Definition of ulcer No. of active ulcers Site of index ulcer Duration of index ulcer Type or classification of index ulcer (where appropriate) Area and depth of index ulcer Presence or absence of infection Infection type (using IDSA or PEDIS grading): none, mild, moderate, or severe Preceding antimicrobial use (type, route, duration, and time before presentation) Involvement of bone or joint Description of how samples were obtained for microbiological examination Type of and results of microbiological examination (Gram stain and susceptibility)
Process	
Technical process	All interventions: details of interventions For each intervention, sufficient information should be provided to define its nature (including source); route (administered systemically, regionally, or topically); frequency; duration of delivery; footwear: details on design, customization, and materials used; evidence of pressure-reducing efficacy if the study relates to plantar ulceration; details on nonsurgical devices, application method, material use, and frequency of replacement; specific design details of the foot-device interface; surgery: details of surgical intervention; surgery undertaken before or in association with antimicrobial administration; open, endovascular, or hybrid revascularization procedures; antimicrobial regimen: route of delivery, agents, and duration; any other relevant intervention (including wound debridement, cleansing, and antiseptic use) undertaken before or in association with antimicrobial administration
Interpersonal process	Person or team administering the delivery of care: professional, nonprofessional carer, the patient Education or behavioral change: whether aimed at patients, carers, or health care professionals Person applying the device: the patient, a non-professional carer, or a health care professional
Outcome	
Foot and limb	Ulcer healing (defined according to existing guidelines; eg, IWGDF)—the number or percentage of index ulcers healed by a fixed time, time to healing, or both Healing following local surgery, including operative debridement First-ever ulcer Recurrent ulcer (specified as being at the same site as a previous ulcer) or ulcer at a different site Amputation level Indication The rationale for amputation level Amputation definition No. of patients/extremities/procedures (re-amputation) High-low amputation ratio Adherence to the intervention (eg, wearing footwear, self-care, education—preferably measured objectively) Adherence to the use of nonsurgical removable interventions Foot pressure reduction (following provision of footwear, surgical interventions, or both) Evidence of pressure-reducing efficacy if the study is on plantar ulceration Ambulatory activity level (for footwear studies), expressed as quantitatively as possible False-positive and false-negative outcomes (in diagnostic self-care studies) Failure to heal by a fixed time Resolution of infection (which should be defined: Texas, SINBAD, PEDIS, or WIfi) at a prespecified time after stopping antimicrobial treatment Clinical or laboratory signs of persistent infection at the end of antimicrobial treatment Days of antimicrobial use, antimicrobial-free days, and days of hospital admission Prevalence of antimicrobial resistance after treatment
Person	Survival Being ulcer-free, amputation-free, or both, at a fixed time after presentation Ulcer-free survival, days Adverse events, adverse device effects, or both No. of participants alive with an intact foot Description of outflow in the foot, in case of surgical or endovascular interventions Measures of the effectiveness of the vascular intervention (eg, toe pressures, TcPo_2_)
Surrogate	Potential surrogate outcome measures for studies in which ulcer incidence is not the primary outcome Incidence of pre-ulcerative lesions (eg, hyperkeratotic tissue, hemorrhage, blister, inflammation—each of which requires definition) Change in ulcer area over a given period Change in ulcer appearance, biochemistry, histology, or other laboratory measure of wound bed status Change in plantar foot pressures Change in adherence Knowledge and behavior (patient, carer, health care professional) Foot examination skill (patient, carer, health care professional)
Patient experience	Patient satisfaction and well-being Health-related quality of life Communication Involvement in decision-making Respect for preferences The overall experience of care received
Patient-centered access to health care	
Patient-centeredness	Patient satisfaction with access to care
Accessibility and equity	Are health care services accessible to all individuals? Geographic proximity of health care facilities Cultural competence of providers
Efficiency and timeliness	Waiting times for appointments Appointment scheduling Waiting times
Effectiveness of care delivery	Ease of navigating the health care system
Barriers to care	Inadequate access to transport/limited time off work Inadequate access to health care providers No stable housing Poverty Insecure employment Not fluent in English/Arabic

Abbreviations: IDSA, Infectious Diseases Society of America; IWGDF, International Working Group on the Diabetic Foot; PEDIS, Perfusion, Extent, Depth, Infection, Sensation; SINBAD, Site, Ischemia, Neuropathy, Bacterial Infection; DepthTcPo_2_, Transcutaneous partial pressure of oxygen; WIFi, Wound, Ischemia, and Foot Infection.

### Data analysis and presentation

The outcomes of the search and the study inclusion process will be extensively documented in the final scoping review and presented in a PRISMA-ScR flow diagram.^36^ Findings will be organized and synthesized according to the predefined review questions.

For qualitative data, thematic analysis will be conducted following Braun and Clarke’s 6-phase framework, which includes familiarization, initial coding, theme development, review, definition, and reporting.^45^ This analysis will be deductive and aligned with each review question (eg, risk factors, barriers to care).

For quantitative data, a descriptive synthesis will be applied. Data will be grouped by health care setting (eg, primary, secondary, or tertiary care). Descriptive statistics (eg, frequencies, percentages) will be reported where possible to summarize key variables such as demographic characteristics, ulcer profiles, and reported outcomes.

Results will be presented using structured summary tables, to depict diabetic foot care pathways, health system differences, and barriers to care. This structured reporting approach enhances transparency and clarity in conveying the methodology and findings of the scoping review.

### Ethics and dissemination

This scoping review will not require ethics approval, because only existing published literature is reviewed. The results will be disseminated at relevant conferences and in peer-reviewed journals.
